# Development of an advanced multiwavelength emission detector for the analytical ultracentrifuge†

**DOI:** 10.1039/d3na00980g

**Published:** 2024-02-19

**Authors:** Vanessa Lautenbach, Georgy Onishchukov, Simon E. Wawra, Uwe Frank, Lukas Hartmann, Wolfgang Peukert, Johannes Walter

**Affiliations:** a Institute of Particle Technology (LFG), Friedrich-Alexander-Universität Erlangen-Nürnberg (FAU) Cauerstraße 4 91058 Erlangen Germany johannes.walter@fau.de; b Interdisciplinary Center for Functional Particle Systems (FPS), Friedrich-Alexander-Universität Erlangen-Nürnberg (FAU) Haberstraße 9a 91058 Erlangen Germany; c Max Planck Institute for the Science of Light Staudtstraße 2 91058 Erlangen Germany

## Abstract

An advanced design of the analytical ultracentrifuge with multiwavelength emission detection (MWE-AUC) is presented which offers outstanding performance concerning the spectral resolution and range flexibility as well as the quality of the data acquired. The excitation by a 520 nm laser is complemented with a 405 nm laser. An external spectrograph with three switchable tunable gratings permits optimisation of the spectral resolution in an order of magnitude range while keeping the spectral region broad. The new system design leads also to a significant reduction of systematic signal noise and allows the assessment and control of inner filter effects. Details regarding the very large signal dynamic range are presented, an important aspect when studying samples in a broad concentration range of up to five orders of magnitude. Our system is validated by complementary studies on two biological systems, fluorescent BSA and GFP, using the commercial Optima AUC with absorbance detection for comparison. Finally, we demonstrate the capabilities of our second generation MWE-AUC with respect to multiwavelength characterisation of gold nanoclusters, which exhibit specific fluorescence depending on their structure. Overall, this work depicts an important stepping stone for the concept of multiwavelength emission detection in AUC. The MWE-AUC developed, being to our knowledge the first and sole one of its kind, has reached the development level suitable for the future in-depth studies of size-, shape- and composition-dependent emission properties of colloids.

## Abstract 2

An advanced multiwavelength emission detector for the analytical ultracentrifuge is presented which offers improved spectral resolution, reduced systematic signal noise, and increased dynamic range for studying samples in a broad concentration range.

## Graphical abstract

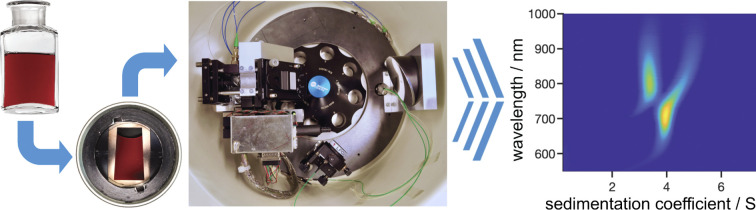

## Introduction

Characterisation methods play a pivotal role in unravelling the intricate nature of complex colloids, offering vital insights into their behaviour and properties. In the realm of nanotechnology, pharmaceuticals, and material science, understanding the structure, composition, and interactions of these systems is paramount for their knowledge-driven design.

In this regard, analytical ultracentrifugation (AUC) stands out as a particularly powerful technique for multidimensional colloid characterisation. AUC capitalises on the principles of sedimentation and diffusion in a centrifugal field to analyse the distributions of mass, size, and shape anisotropy of particles and macromolecules in solution with outstanding accuracy and reproducibility. The versatility of AUC, offering a comprehensive view on the structural heterogeneity of a system, is highlighted by its applicability to a wide variety of colloids including nanoparticles,^1–3^ polymers,^4–6^ and biomolecules.^7,8^ Its ability to discern minute variations in particle properties and interactions makes it invaluable for unravelling the complexity inherent in these systems. Moreover, having the sample in the native solution environment preserves the colloid's integrity and minimises artefacts, which can arise from sample preparation.

In the past 20 years, AUC experienced a significant boost for its application in characterising colloids with increasing complexity by implementing new hardware and data analysis tools. Modern AUCs can feature wavelength-resolved absorbance, interference as well as integral fluorescence detection. In addition, a multiwavelength absorbance detection system has significantly extended capabilities for multidimensional characterisation of nanoparticles and macromolecules.^4,9–15^ Recently, our group presented a multiwavelength emission detector (MWE-AUC),^16,17^ which permits measurements of sample fluorescence spectra as a function of the sedimentation coefficient. For the first time, the fluorescence features could be directly linked to the sedimentation coefficient and in this way to the particle size. This first generation (Gen1) MWE-AUC setup featured a miniature spectrometer with a fixed spectral resolution and excitation at 518 nm only. A critical downside of the Gen1 MWE-AUC was the presence of some excess signal variations. While such systematic noise contributions can in principle be corrected *via* dedicated algorithms in data analysis software such as SEDFIT,^18,19^ it hampers possibilities for multiwavelength analysis due to the necessity to perform such corrections using additional fit parameters for each wavelength, which is computationally expensive.

In this paper, we report on the design of a second generation (Gen2) MWE-AUC setup with improved performance further extending its applicability for investigations of complex systems, which require high spectral resolution and straightforward multiwavelength analysis. First, the hardware developments concerning the optical and mechanical design, excitation laser options, sample fluorescence detection, and new sample cells as well as extended software features are considered. New aspects of system alignment and calibration are also discussed. Next, the characterisation of the Gen2 MWE-AUC performance especially concerning signal linearity and noise, dynamic range, and the possibility to control inner filter effects are reported. Using fluorescein iso-thiocyanate conjugate of bovine serum albumin (F-BSA) and green fluorescent protein (GFP) as two representative macromolecules, results of sedimentation velocity experiments are validated by complementary measurements using the Optima AUC as a commercial machine with the absorbance detection system. Finally, we demonstrate the efficiency of the MWE-AUC for the characterisation of gold nanoclusters (AuNCs) with respect to their hydrodynamic and fluorescence properties.

## Materials and methods

### Fluorescent dye coumarin 153

Coumarin 153 (catalogue #546186) purchased from Sigma-Aldrich (St. Louis, Missouri, United States) was dissolved in absolute ethanol (≥99,8%, VWR, Darmstadt, Germany). A stock solution with a concentration of 0.1 g L^−1^ was prepared and further diluted by addition of ethanol.

### Fluorescein iso-thiocyanate conjugate of bovine serum albumin (F-BSA)

F-BSA (catalogue #A9771) from Sigma-Aldrich (St. Louis, Missouri, United States) was used without further purification. F-BSA was dissolved in a 12 mM tris and 15 mM NaCl buffer up to a final concentration of 9.0 μM and further diluted by a factor of 1 : 20 to 0.45 μM.

### Green fluorescent protein (GFP)

GFP (catalogue #14-392), a recombinantly produced protein expressed in *E. coli* from Merck KGaA (Darmstadt, Germany), was used as delivered with a purity of >70%. The formulation contains phosphate-buffered saline (PBS) and 20 v% of glycerol. For absorption measurements, the purchased protein suspension was diluted to a concentration of 5.0 μM using a 20 v% glycerol/PBS solution. For evaluation measurements in the MWE-AUC, this sample was diluted 1 : 2 by addition of the glycerol/PBS buffer to a concentration of 2.5 μM. Prior to further dilution, a 1.5 μM bovine serum albumin (BSA) (catalogue #A7030) from Sigma-Aldrich (St. Louis, Missouri, United States) solution was prepared diluting the BSA in PBS buffer. For concentrations in the nanomolar range, GFP was diluted according to the protocol of Chaturvedi *et al.*^20^ using the BSA/PBS buffer to avoid uncertainties in GFP sample concentration due to potential surface adsorption.^20^

### Gold nanoclusters (AuNCs)

The synthesis of glutathione-capped AuNCs was performed as described previously.^21^ In brief, a mixture of 5 mM NaAuCl_4_ and 20 mM glutathione in 12.5 mL methanol was prepared and stirred for 30 min at room temperature, subsequently cooled to 0 °C and then stirred for another 30 min. An aqueous solution of 150 mM NaBH_4_ (3.13 mL, cooled to 0 °C) was then rapidly injected into the methanol solution under vigorous stirring. The mixture was allowed to react for 3 h after which the synthesised AuNCs were centrifuged, washed three times with methanol and dried under ambient conditions. For MWE-AUC experiments, a particle suspension was prepared in 100 mM NaCl with a concentration of 1 g L^−1^.

### Optima AUC

An analytical ultracentrifuge from Beckman Coulter, type Optima AUC, was used to validate the Gen2 MWE-AUC results by performing comparative measurements with the proteins detailed above. The absorbance detection system was used at 398 nm and 495 nm for GFP and F-BSA, respectively, which according to preceding UV-VIS measurements corresponds to the absorbance maxima of the samples (*cf.* ESI, Fig. S3†). Sedimentation velocity measurements were performed at a rotor speed of 40 000 rpm and 20 °C using titanium centrepieces with a path length of 12 mm. Scans were taken every 90 seconds with 450 scans recorded in total.

### MWE-AUC

A modified preparative ultracentrifuge from Beckman Coulter, type Optima XL-80K, equipped with the custom-made fluorescence detection setup described in this study was used for the experiments. Details about the equipment used can be found in our previous publication on this device^16^ and in this manuscript. The excitation wavelength used for the studies was either 405 nm or 520 nm. Prior to the AUC experiments, the sample concentrations were controlled by UV-VIS measurements (*cf.* ESI, Fig. S4 for coumarin 153 and Fig. S7† for GFP). All measurements were performed at a temperature of 20 °C using custom 3 mm titanium mono-sector cells described in more detail in the subsection “Mono-sector cells with 3 mm centrepiece” of this manuscript. The radial scan step was 50 μm in all studies; details about the rotor speed and other specific parameters are provided in the text.

### Data analysis

SEDFIT (version 16.1) was used to derive the continuous c(s) distributions for F-BSA and GFP.^18^ The fluorescence signal amplitude was re-scaled to a maximum below 10 prior to data analysis in SEDFIT. Fluorescence-specific signal corrections accessible through the toolbox in SEDFIT were not applied during analysis of data from the Gen2 MWE-AUC because corrections beyond radial- and time-invariant noise treatment are not implemented in our custom analysis software for multiwavelength data, and we aimed for a comparable data analysis for the fluorescence and absorbance measurements. SEDFIT standard parameters (partial specific volume = 0.73 cm^3^ g^−1^, buffer density = 1.0 g cm^−3^, buffer viscosity = 0.01002 poise) were used for the evaluation of the F-BSA and nanomolar GFP protein samples. For the GFP samples in the micromolar range, the density and viscosity of the solution was calculated following the protocol from literature.^22,23^ The final solution parameters are provided in the figure descriptions. Radial- and time-invariant noise and the meniscus position were fitted for all measurements. For evaluation of the protein data, the frictional ratio was treated as a floating parameter and the confidence level for the maximum entropy regularisation was set to 0.683. A sedimentation coefficient resolution of 150 values was used for all analyses.

Assuming the AuNCs to be of spherical shape, the continuous c(s) model with fixed frictional ratio of 1.0 and floating partial specific volume (starting the fit with a value of 0.4) implemented in SEDFIT was used for the evaluation. The confidence level for the second derivative regularisation was set to 0.683. The fitted parameters were then used as input for the 2D analysis to speed up calculations in our custom code. The best-fit partial specific volumes were 0.547 cm^3^ g^−1^ and 0.63 cm^3^ g^−1^ for excitation at 405 nm and 520 nm, respectively. For the 2D analysis, we performed consecutive c(s) analyses for all wavelengths rather than just for certain wavelengths as done in SEDFIT based on our previous work^24^ using the algorithms developed by Schuck *et al.*^18,25^ Regularisation was performed using the second derivative and a confidence level of 0.683. The partial specific volume was taken constant for all wavelengths. A sedimentation coefficient resolution of 150 values was used, and the wavelength step was 1.5 nm. For analysis in SEDPHAT (version 14.0), we used the “Species Analysis” model and performed a single fit prior to the global fit (including floating meniscus and time as well as radial noise fitted).

## Hardware and software developments

**Fig. 1:**
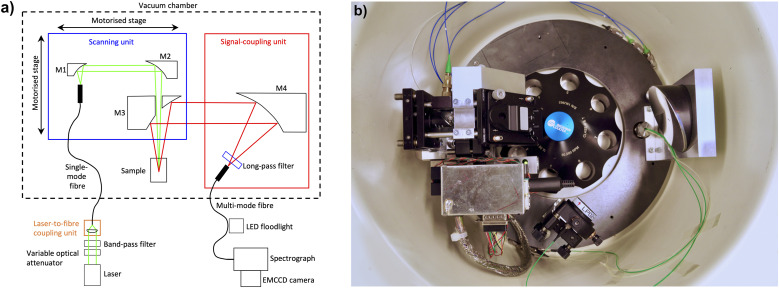
(a) Main components of the revised optical scheme of the Gen2 MWE-AUC projected on a single plane. (b) Photograph of the Gen2 MWE-AUC setup implemented in the vacuum chamber of the Optima XL-80K ultracentrifuge with optics, fibers, and electronics distributor box.

Several modifications for the Gen1 MWE-AUC have been made since our previous publication^16^ to improve its performance concerning the spectral resolution and range as well as the quality of the data obtained. The most important developments of the hardware setup are described in the corresponding subsections below. The revised optical scheme and a photo of the setup are depicted in Fig. 1. A new list of optical, mechanical, and electronic components is given in the ESI.†

### Mechanical design

As the signal amplitude strongly depends on the overlap of the light spots in the excitation and fluorescence signal paths in the sample, the Gen1 MWE-AUC has been found to be quite sensitive to mechanical vibrations of the optical components, especially of the mirror M2 in Fig. 1a. Such vibrations are mainly transferred from the chamber body to the scanning unit by the steel ring, which serves as the baseplate for the optics inside the chamber. In particular, we have observed significant excess signal noise when mechanical resonances are excited at certain rotation speeds and some additional noise when the pre-vacuum pump starts running. To eliminate these effects, the oil pump was placed on sorbothane vibration absorbers outside the machine and the steel ring was only firmly fixed on the three spacers using PTFE washers under the ring and springs above, which are placed between the ring and the screw heads.

These modifications proved to successfully dampen vibration-inducted fluctuations of the detected fluorescent signal. Furthermore, no light-spot misalignment due to steel-ring deformation under vacuum has been observed now using a miniature wireless camera; but a 2 mm shift of the focal plane in z-position is still apparent under operation conditions. This effect is caused by the warping of the heat sink when vacuum is applied.^26^ However, as the chamber deformation is well reproducible, it can be easily compensated by tuning the z-stage. To simplify optimisation of the z-position of the confocal plane in the sample and, in consequence, of the signal collection volume under operating conditions, the manual translation z-stage of Gen1 MWE-AUC has been replaced by a motorised stage with computer software control. This allows to perform z-scans during rotation in vacuum for all samples in the rotor, which showed to be very important for control and prevention of inner filter effects (for details it is referred to the section on the inner filter effects below). Usually, all the cells in the rotor have very similar z-position of the sample if the same cell components, *i.e.*, housings, centrepieces, windows, are used. Nevertheless, if necessary, the z-position can be individually adjusted for each cell in the rotor.

### Excitation laser sources

While the Gen1 MWE-AUC featured a single green 518 nm laser with analogue amplitude modulation, either one of the two diode lasers, a blue one at 405 nm and a green one at 520 nm, both with digital current modulation can be used in the Gen2 MWE-AUC. Digital modulation enables generation of light pulses with very short, <10 ns, fronts and high extinction ratio. In consequence, longer excitation pulses providing stronger fluorescence signals can be used, while minimising signal contributions from the centrepiece sidewalls and cell housing at the same time.

Changing of the excitation wavelength can be performed by reconnecting the single-mode fibre between the two laser-to-fibre coupling units, each one optimised for a particular laser, and by placing the corresponding spectral long-pass filter before the multi-mode fibre in the signal-coupling unit. Usually only optimisation of the laser-to-fibre coupling and neither realignment nor radial recalibration are necessary; but at least a check of the beam overlap is recommended.

To minimise the contribution of the broadband background (amplified spontaneous emission from the laser diode chip) in the spectral region close to the cut-off of the long-pass filter in the signal-coupling unit, the lasers are normally operated at a constant high current of 150 mA. A manually tuneable neutral-density disk filter before the fibre-coupling unit is then used as a variable optical attenuator for optimisation of the excitation light power. If necessary, computer software control of the laser diode current can also be used to control the excitation power for the cells in the rotor individually. Two additional spectral band-pass filters have been installed for laser-line cleaning before the fibre-coupling units, which can also be placed between the mirrors M1 and M2 in the scanning unit, if it becomes necessary to suppress also the fluorescence or Raman scattering in the single-mode fibre.

### Optics, confocal characteristics, and alignment procedure

In addition to the variable optical attenuator for the laser power optimisation and additional laser-line cleaning filters mentioned above, an important modification of the setup optics in the Gen2 MWE-AUC is the use of a multi-mode fibre with 200 μm core diameter instead of 50 μm and the same numerical aperture of 0.22. This allows an increase of both the spot size and the depth of the confocal volume in the sample, from which the fluorescence signal is collected. With the 50 μm core fibre in the Gen1 MWE-AUC, the confocal spot size and the diameter of the laser beam were about the same (∼35 μm, depending of the laser wavelength). In consequence, a small laser beam misalignment of just a few μm led to a drop of the signal amplitude just as observed for the vibration-induced oscillations of the optics. In addition, because of imperfections of the translation stage used for radial scans, a change of its moment load causes a small monotonic drift of the laser beam during radial scans, which results in radius-dependent, time-invariant variation of the fluorescence signal. Depending on the initial beam alignment, a beam drift can lead to an increase or decrease of the fluorescence signal with radial position, or even formation of a local maximum. Such effects can partially be accounted for by data analysis tools such as SEDFIT^19^ but are rather difficult to deal with when aiming for multiwavelength analysis as additional fit parameters will require extensive computing resources.

The use of a 200 μm core fibre in Gen2 MWE-AUC increases the signal confocal spot size to 135 μm, by a factor of 4, while the size of the laser beam remains the same. In this case, the laser beam spot remains within the signal confocal spot even for drifts of about 50 μm and coupling of the fluorescence signal into the fibre does not change assuring strongly reduced radius-dependent, time-invariant signal distortions during radial scans. Furthermore, the large fibre core size increases the confocal depth from about 0.7 mm to 1.5 mm making it comparable with the depth of the 3 mm measurement cell. In consequence, the fluorescence signal from nearly the full sample volume is collected rather than only from a part of it, which leads to a corresponding increase of the signal amplitude.

As the excitation-laser beam profile, being close to the fundamental mode for both 405 nm and 520 nm lasers, has very low divergence (estimated Rayleigh length ∼2 mm), its spot size is very similar over the depth of the confocal volume in the sample. Hence, the radial resolution of ∼40 μm remains practically the same as for the old small-core fibre used in the Gen1 MWE-AUC, because the radial resolution is defined now by the excitation laser spot diameter only rather than by the convolution of the two beam spots, one having a flat-top profile (*cf.* ESI, Fig. S5†). Notably, this is quite different from the fluorescence-detection system (AU-FDS),^27,28^ where the numerical aperture of the excitation beam and of the fluorescence signal collection are similar and determine together the radial resolution.

Periodical control of optics alignment can be performed in a straightforward manner by using an additional common camera at the cell position in the AUC chamber and a dedicated light source. Instead of an additional laser pointer coupled in the multi-mode fibre from the spectrograph side to simulate back-propagation of the fluorescence signal, just a common 20 W white LED floodlight illuminating the bent fibre in the transparent 900 μm buffer from the side can be used without need of any intervention to the fibre connections. Light intensity coupled mainly in the cladding modes is quite low but still sufficient for a complementary metal oxide semiconductor (CMOS) camera with exposure times of about 200–500 ms. Accordingly, the power of the excitation laser must be strongly reduced by setting its current to just a few mA in the CW operation mode. The large sensor size of the CMOS camera used to observe the beams in the focal plane allows the control of the beam spot overlap in the full radial scan range. Mounting the CMOS camera on a manual translation stage enables the check of the beam shape along the *z*-axis in a few mm region. Control of the beam overlap is strongly recommended each time the scanning unit has been accidently touched during rotor installation in the chamber.

### Spectrograph and camera for fluorescence detection

The spectral resolution and the spectral range are extended in the Gen2 MWE-AUC by using a mid-focal length imaging spectrograph with an adjustable entrance slit and three switchable, tuneable gratings (150, 600, and 1800 L mm^−1^) on an exchangeable turret in combination with a scientific-grade electron-multiplying charge-coupled device (EMCCD) camera. In contrast, the Gen1 MWE-AUC was equipped with a miniature spectrometer with a fixed grating and slit in combination with a CCD array detector. Grating tuning is very useful to optimise the laser-line position near the blue end of the spectral range, while different gratings allow measurements either in a broad spectral range covering >500 nm, *e.g.*, 400–937 nm, with 2.0 nm spectral resolution using the 150 L mm^−1^ grating, in a medium spectral range covering >100 nm, *e.g.*, 515–643 nm, with 0.51 nm spectral resolution using the 600 L mm^−1^ grating, or in a narrow spectral range covering >30 nm, *e.g.*, 525–558 nm (1125 cm^−1^), with 0.14 nm resolution using the 1800 L mm^−1^ grating. High spectral resolution leads to a proportional decrease of the signal amplitude as the signal spectral density remains the same. In the case of high spectral resolution, it is still possible to study a broader spectral range by either tuning the grating or even changing the gratings between repetitive scans in sedimentation equilibrium and, under certain conditions, in sedimentation velocity experiments.

A multi-mode fibre couples light into the spectrograph, which is positioned outside the vacuum chamber, as before in the Gen1 MWE-AUC. The width of the entrance slit, located right behind the fibre output facet, is usually optimised for a minimal decrease of the signal amplitude but can be made smaller to improve the spectral resolution at the expense of a signal amplitude decrease. The EMCCD camera with 1600 × 200 pixels is usually operated in the full vertical binning mode providing spectral data with 1600 points. When no multiwavelength characterisation is necessary, and just the total fluorescence signal is to be measured similar to the AU-FDS, binning along both dimensions over the pixel area, covering the full signal spectrum on the sensor chip, is possible. Due to a higher quantum efficiency of the EMCCD camera (>80% in the 420–800 nm region *vs.* ∼40–20% for best photomultipliers) and the low binning and read noise, this approach can lead to similar or even better sensitivity performance compared to the AU-FDS system with a spectral filter and a photomultiplier detector.

The signal dynamic range of the 16 bit analogue-to-digital converter (ADC) can be additionally extended by switching the camera electronic gain from 1 electron per count to 4 electrons per count. The sensor-internal electron multiplication is not usually used because it hardly improves the signal-to-noise ratio (SNR) of a reasonably strong signal when the read noise is negligible and rather makes it worse by the excess multiplication noise. Deep sensor cooling allows exposure times up to 500 ms without an increase of signal background offset by the dark current signal above the noise level. The camera is usually operated in the exposure trigger mode. To avoid sensor illumination outside the camera exposure time window, the train of laser trigger pulses is additionally gated by the camera exposure signal using simple custom-made electronics (*cf.* ESI, Fig. S1 and S2†).

### Data acquisition software

The data acquisition (DAQ) software written in Labview was developed further based on the Gen1 MWE-AUC version to make use of the new hardware developments. For sedimentation velocity experiments, samples are now scanned consecutively which allows the setting of sample specific exposure times and laser intensities. The signal integration time, during which the sample is excited by the laser, is automatically adjusted depending on the rotor speed, and the camera exposure time can be adjusted to accumulate the signal over a number of rotations for each cell channel individually, which permits the measurements of samples with very different concentrations in a single experiment. Notably, for a given camera exposure time, the signal amplitude remains the same for different rotation speeds if the duty cycle of the laser pulses remains constant. In addition, the new DAQ software features functionalities for all calibration procedures described in the next section.

## Measurement cells and calibration

### Mono-sector cells with 3 mm centrepiece

**Fig. 2:**
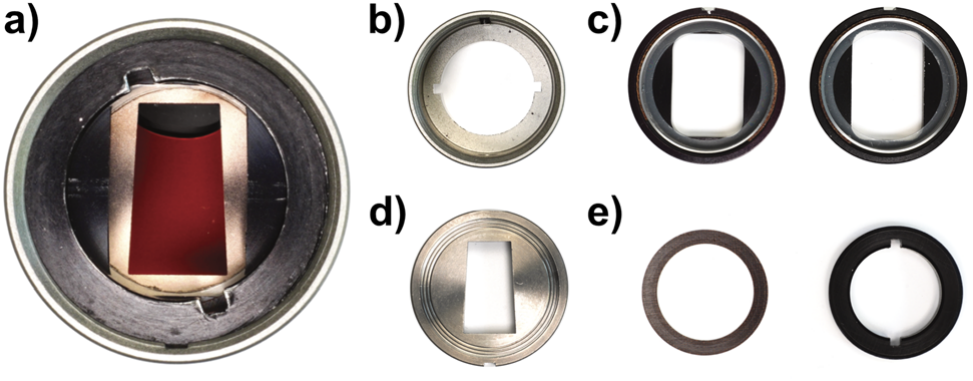
(a) Assembled measurement cell with custom-made 3 mm titan mono-sector centrepiece for improved chemical resistance, less sample volume, and higher integration times. (b–e) Cell components: (b) housing, (c) sapphire windows including window housing, window liner, and window gasket, (d) centrepiece, (e) screw ring gasket and screw ring.

So far, we only had access to 12 mm mono-sector epoxy and aluminium centrepieces, originally designed and manufactured for the Schlieren detection system.^29^ While such centrepieces can be used for most aqueous dispersions, their applicability for organic solvents is limited. Therefore, the Gen2 MWE-AUC applies 3 mm mono-sector titania centrepieces, designed together with and manufactured by Nanolytics Instruments (*cf.*Fig. 2), which grant highest chemical compatibility and reduce the amount of sample needed. Besides, our setup can be operated with standard 3 mm or 12 mm double-sector centrepieces, which doubles the number of samples in the rotor, but accordingly reduces the signal amplitude due to shorter signal integration times. Alternatively, custom 3D-printed centrepieces can be used.^30^

### Radial and angular calibration

Instead of the two-point radial calibration using the counterbalance with neither glass windows nor sample solution in the Gen1 MWE-AUC, a more precise multi-point calibration using a cell with a calibration disk^31^ on the top of a 3 mm thick disk of the fluorescent acryl glass 2C01 is now used. The glass is chosen instead of a liquid dye solution because of better long-term stability as well as to avoid contamination of the calibration disk by the dye.

The step size and movement linearity of the step motor, providing the radial scan, and the absolute radial position can be evaluated using the known gap layout of the calibration disc. The estimated calibration accuracy of the absolute radial position is ∼100 μm, which is determined mainly by the accuracy of the positioning of the calibration disk in the cell housing. Still, related positioning errors are insignificant as the error in the determined sedimentation coefficient will be <0.2%. Overall, step size and movement linearity are much more accurate.

**Fig. 3:**
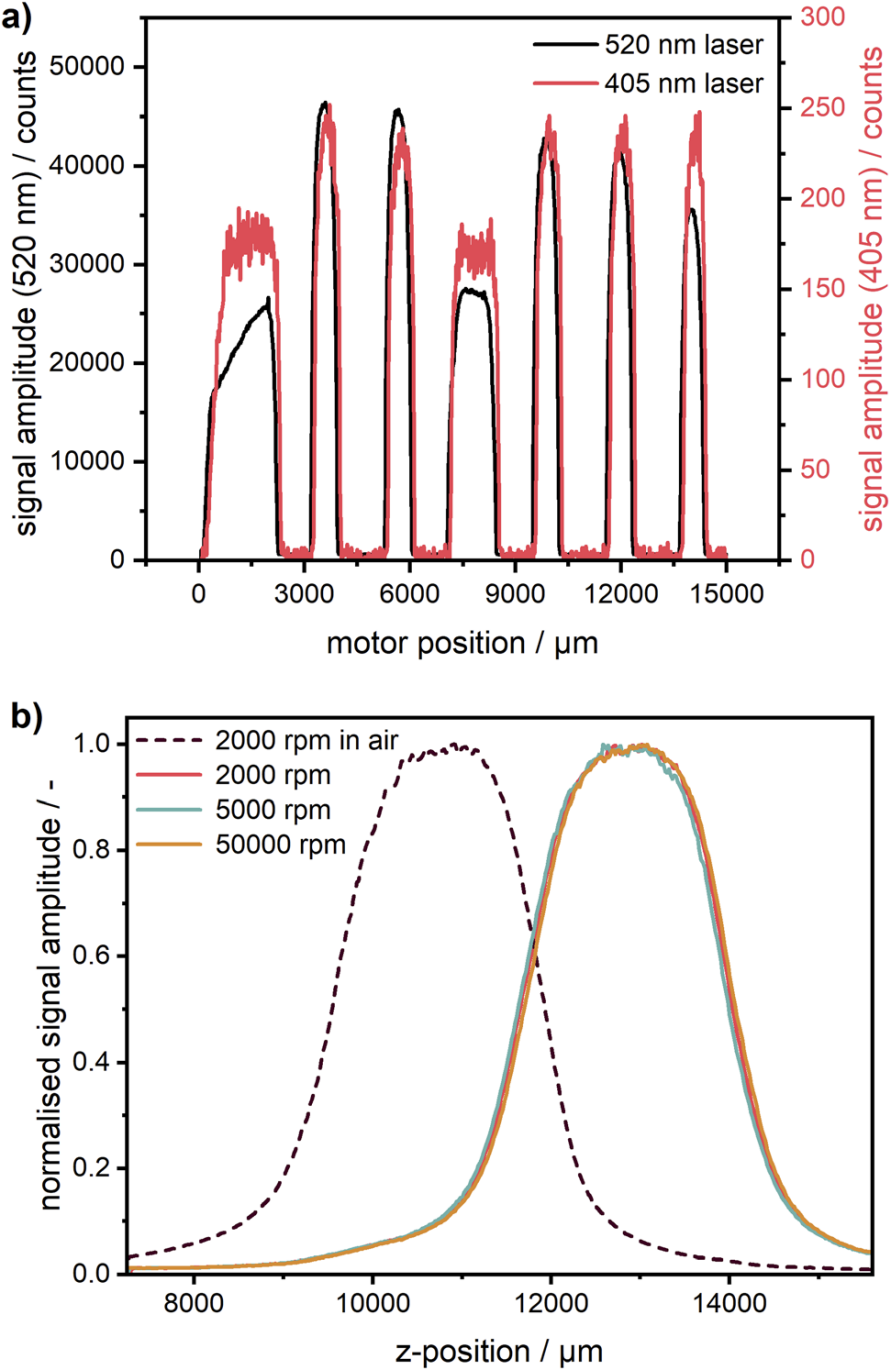
(a) Radial scans of the cell with the calibration disk for two different excitation wavelengths at the respective emission maxima. (b) Z-scans of coumarin 153 (1 mg L^−1^) in ethanol at 10 ms exposure time at different rotation speeds in vacuum. The sample was excited with the 405 nm laser. A 10 times signal averaging was applied to improve the data quality.

The calibration cell was scanned with both 405 nm and 520 nm excitation lasers (*cf.*Fig. 3). The step size is practically identical, no non-linearity due to imperfections of the translation stage can be observed. A radial shift of ∼80 μm can be observed, which most likely is a consequence of optics realignment in a timespan of three months between the measurements.

Using this type of calibration, such potential radial shifts can be easily compensated for. As can be seen in Fig. 3a, the fluorescent acryl glass of type 2C01 provides a much weaker signal for the 405 nm laser because it was selected earlier for green excitation only. Another glass type, better suitable for multiwavelength operation, can be used if necessary.

### Calibration of the z-coordinate

Due to the automated z-stage, it is possible in our Gen2 MWE-AUC to control the z-position of the signal focal plane similar to the AU-FDS.^27,28^ It is a part of the workflow to check the sample concentration as well as the optimum z-position of the cells measured. This is an important aspect with respect to the inner filter effects, which can occur at too high concentrations (see section on the inner filter effects). As shown in Fig. 3b, a significant difference in cell position can be observed under ambient conditions and in vacuum. Under atmospheric conditions, the signal maximum and respectively the centre of the sample is at 10.9 mm. After applying the vacuum, the cell centre shifts up by 2.0 mm in z-position and can be found at 12.9 mm. This is caused by warping of the heat sink which changes the cell position relative to the steel ring with the mounted detection optics. Therefore, the z-position, at which measurements are subsequently taken, needs to be determined only after the vacuum is established. As expected, the comparison of the z-scans at different rotational speeds shows that the cell position remains constant across different rotational speeds. In the case of very fast sedimenting samples, when an optimisation is not feasible even at the lowest rotor speed of 1000 rpm, this preparatory measure can also be performed at 0 rpm by using a pulse generator as a rotor trigger source. Thereby, the sample sealing can be checked, and the excitation laser power can be optimised under vacuum without any rotation, while the system operates in the same way as during measurements. Obviously, this procedure can be performed under vacuum only for a single cell. If a check of multiple cells is necessary, the chamber has to be opened and the rotor angle must be readjusted for the next cell. As our experience shows, the preliminary check of multiple cells can frequently be performed shortly already under ambient conditions with an open chamber taking into account the corresponding shift of the cell z-position.

## Experimental performance assessment

### Signal-concentration linearity using dye samples

**Fig. 4:**
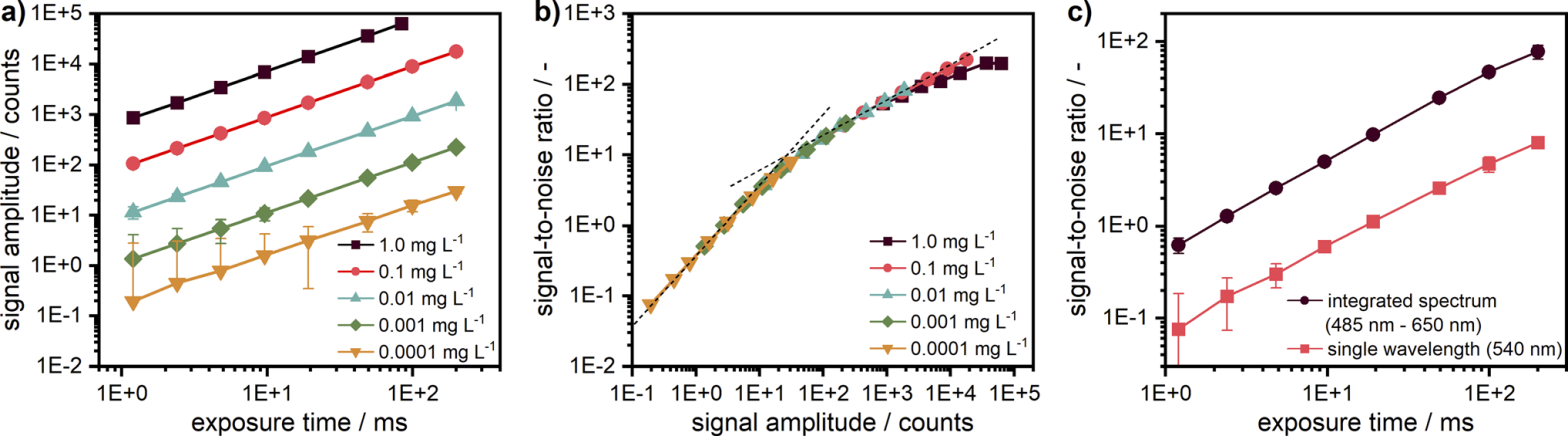
(a) Linearity check for five target concentrations of coumarin 153 diluted in ethanol. The signal intensity is given for the fluorescence at 540 nm. (b) Signal-to-noise ratio (SNR) over signal intensity for the target concentrations at 540 nm; the two dash lines indicate slopes of 1 and 0.5. Negative error bars are not shown in the logarithmic representation. For better illustration, the first data points of the two lowest concentrations at small exposure times can be found in the ESI† as a linear plot of the signal intensity (*cf.* Fig. S6†). (c) Comparison of the SNR between measured data for a single wavelength (540 nm) and integrated spectrum (485–650 nm) for the lowest target concentration (0.0001 mg L^−1^).

For the measurements, it is very important to ensure that the fluorescence signal recorded is linearly proportional to the sample concentration to avoid further fitting parameters during data analysis. The non-linearity could be caused by several effects, amongst which the most critical are the inner filter effects and the detector background offset. According to specifications, the linearity of the used EMCCD camera is better than 99%. The background offset mainly stems from the electronic offset, which is very stable and can be subtracted prior to the experiment, while for camera exposure times below 500 ms, the dark current is sufficiently small to be neglected. The linearity was checked for five target dye concentrations of 1.0, 0.1, 0.01, 0.001, and 0.0001 mg L^−1^ obtained by subsequent dilution by a factor of 10. Each sample was measured at different exposure times of the camera. As one can see in Fig. 4a, the linearity could be confirmed for the entire investigated concentration range spanning four orders of magnitude and more than five orders of magnitude for the signal amplitude.

The intensity-dependent relationship between SNR and signal amplitude is shown in Fig. 4b. The overlapping of the SNR values for different concentrations points out the excellent detector linearity. As expected, the SNR is linearly proportional to the signal amplitude for low counts (<10) being mainly determined by the camera read noise there. It becomes proportional to the square root of the signal amplitude at high counts (>100), when shot noise becomes the main noise component. At high signal amplitudes, it is important to avoid detector saturation by noise spikes.

For low concentrations or low exposure times, the signal becomes weaker and therefore noisier. Hence, the SNR can be improved either by increasing the camera exposure time up to ∼250 ms or even 500 ms or by averaging over several acquisitions as employed for the AUC with multiwavelength extinction detection, which takes more time due to additional digitalisation and data transfer cycles and is less efficient due to an increase of the overall read noise. If a further increase of the camera exposure time is not possible, *e.g.*, due to detector saturation, additional averaging can be used but will lead to an increased measurement time, which can be problematic for sedimentation velocity runs but still applicable for sedimentation equilibrium experiments.

By virtue of the multiwavelength capabilities of our system, if similar to the AU-FDS high spectral resolution is not necessary, then the signal data can be integrated across different wavelengths to increase the SNR. As shown in Fig. 4c, an improvement in SNR by about a factor of 10 can be achieved for the lowest concentration by data integration over 487 sensor pixels in the spectral range of 485–650 nm (nearly full fluorescent band of the dye).

An improvement of the SNR for weak signals while keeping the spectral resolution is also possible by changing the ADC sampling rate from 1 MHz to 100 kHz, which results in a read-noise reduction from 7 electrons to 4 electrons rms, according to the camera specifications, at the expense of an increase of the read time from 1.6 ms to 16 ms.

### Inner filter effects

Similar to the absorption technique, the MWE-AUC requires the sample to be in a suitable concentration range in order to exclude non-linearities in the signal dependence on sample concentration during sedimentation. For too low concentrations, the fluorescence signal is weak and has low SNR. For too high concentrations, besides the saturation of the detector, which can be reached even at very short camera exposure times corresponding to a single laser pulse, so-called inner filter effects^32–35^ can disturb the measurement results for fluorescence-based AUC.^28,36^ These effects occur at elevated sample concentrations, if the excitation laser light is already absorbed so strongly in the upper layers of the cell due to a too high extinction that the signal excitation at the bottom or even centre cell layers is considerably weaker – this is also referred to as the primary inner filter effect.^37^

In addition to the excitation-related inner filter effect, such disproportionalities can also be caused by self-absorption of the fluorescence, the secondary inner filter effect.^38^ This occurs usually on the blue side of the fluorescence spectrum when the absorption and emission bands of the sample overlap (*cf.* ESI, Fig. S7†). The secondary inner filter effect is typically much weaker than the primary inner filter effect and can therefore be observed especially, when the extinction at the excitation wavelength is comparable or even weaker than that for the fluorescence, which is usually the case when the excitation is far aside of the absorption peak. Then, the light emitted from the depth of the sample can be reabsorbed before leaving the cell, which weakens the detected signal. Care should be taken especially for complex colloids where the absorption and fluorescence bands of different components might overlap.

As sedimentation results in changes of the local concentration, mainly dilution with sometimes a significant increase at the bottom due to back-diffusion, the relation between concentration and signal intensity might become quite non-linear due to the inner filter effects. That makes the interpretation of the measurement data during evaluation difficult or even impossible.

With the motorised z-stage added in the Gen2 MWE-AUC, so-called z-scans can be easily made any time during measurements. Being especially practical to perform prior to the measurement, z-scans make it very easy to determine whether the samples are in a suitable concentration range or not and to set the optimum measuring depth (z-position of the confocal volume in the sample). If the sample concentration is in an appropriate range, a mostly symmetrical signal profile is generated, which has its maximum in the middle of the cell.

**Fig. 5:**
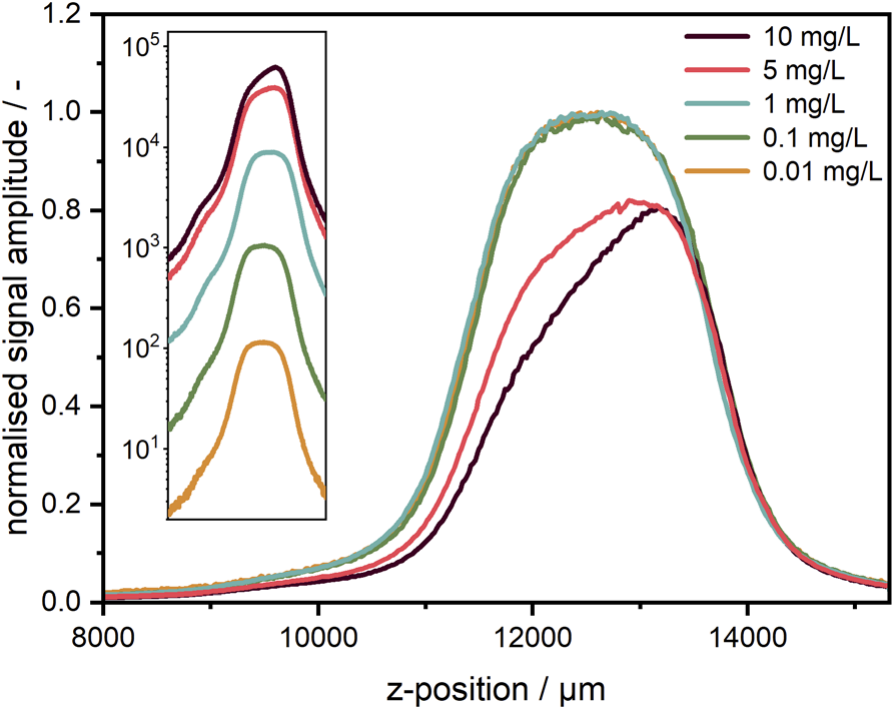
Z-scans of coumarin 153 samples at different concentrations dissolved in ethanol for demonstration of the concentration-dependent primary inner filter effect. Measurements were performed at 5000 rpm and a radius position of 6.6 cm. Data was extracted at 540 nm, which is close to the emission maximum. Inset: the same data are shown on logarithmic scale without normalisation.

However, if the sample is too concentrated, the inner filter effects make the profile of the z-scan asymmetrical and shift its peak (Fig. 5). To avoid this situation, either the sample must be diluted or the signal must be collected from a smaller z-range around or better before the signal maximum (on the maximum's right side in Fig. 5) with corresponding decrease of the signal amplitude. However, this approach can lead to distortions in the measured fluorescence profiles due to slight variations of the z-position of the confocal volume during radial scans, as the measurement takes place then in a region of a relatively strong signal gradient.

The secondary inner filter effect is illustrated in Fig. S8 in the ESI† using coumarin 153 samples excited at 405 nm by z-scans at the wavelengths of 455 nm and 470 nm. For a fixed excitation wavelength, different from the primary inner filter effect, the influence of the secondary inner filter effect can easily be avoided by the proper choice of the fluorescence wavelength.

Furthermore, radial and z-scans of different wavelengths were compared with each other (*cf.* ESI, Fig. S5 and S8†). As already shown by Wawra *et al.*,^16^ wavelength-independence could be confirmed. This allows the determination of useable measurement results for samples with homogeneous spectral broadening even at very high signal intensities that lead to camera saturation at the spectrum maximum by evaluation of the data in a spectral region of a lower signal at a different wavelength.

## Application to macromolecular and particulate systems

For the development of our Gen2 MWE-AUC, constructional, optical, and software modifications were implemented and contribute to a significantly improved data quality. For example, the signal oscillations caused by the vibration of the pre-vacuum pump and mechanical resonances at certain rotor speeds were eliminated. The radial instabilities of the signal observed for the Gen1 MWE-AUC were avoided and performing reproducible z-scans under vacuum is now possible.

**Fig. 6:**
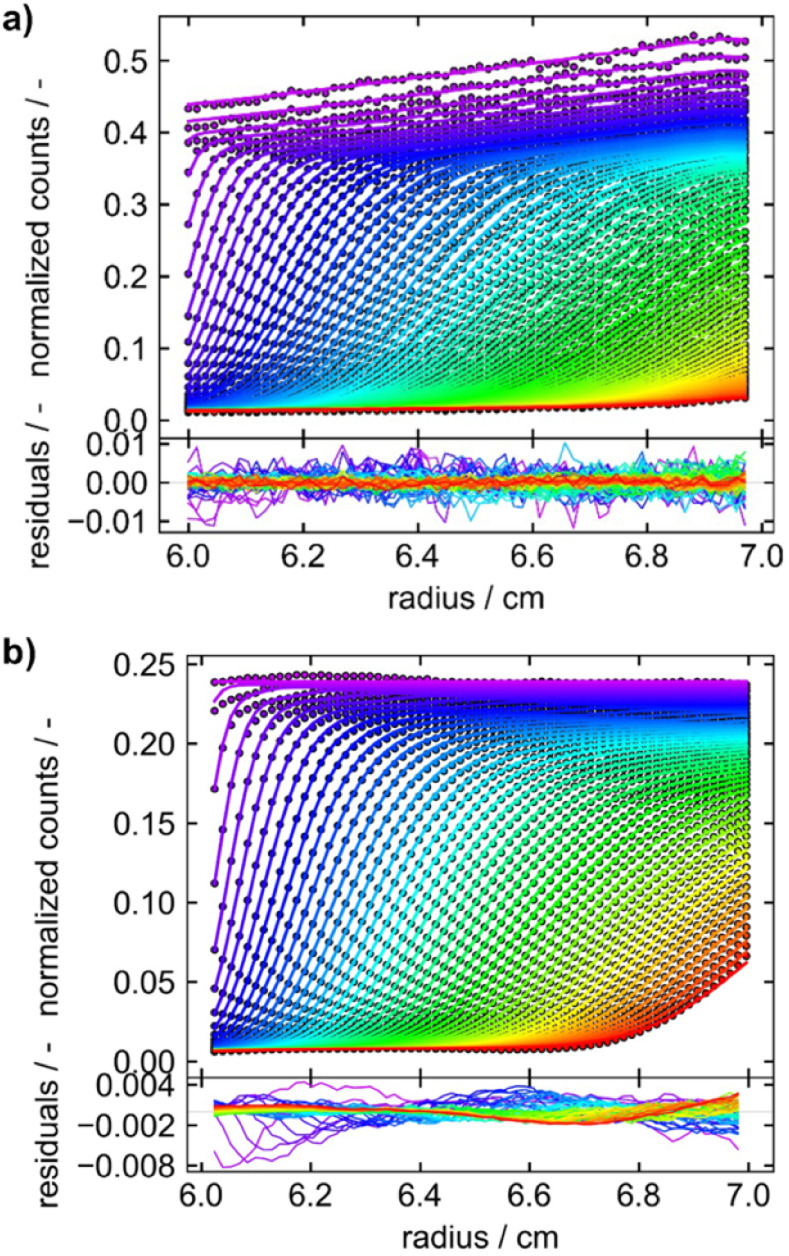
Sedimentation velocity data demonstrating data quality improvement from (a) Gen1 MWE-AUC to (b) Gen2 MWE-AUC. (a and b) show the data of 9.0 μM F-BSA diluted in a 12 M tris and 15 M NaCl buffer, measured at 40 000 rpm. The F-BSA sample was excited at 518 nm and 520 nm and its emission was evaluated at 550 nm and 555 nm for (a and b), respectively. (a) is adapted from Wawra *et al.*^16^ Copyright (2019) Nanoscale Advances. All raw data were analyzed using SEDFIT (version 16.1c) and plotted together with the best-fit profiles and respective residuals using Gussi (version 1.4.2). Data were analyzed with fluorescence corrections switched on and off for the Gen1 and Gen2 data, respectively.

Furthermore, a better wavelength resolution is achieved using a spectrograph with tuneable gratings instead of the miniature spectrometer. Overall, data obtained by the Gen2 MWE-AUC exhibits significantly less, if any time-invariant and fluorescence-specific noise components as can be seen from the direct comparison of both generations depicted in Fig. 6. This opens up opportunities to perform efficient multiwavelength analysis of data as only radial-invariant noise needs to be accounted for during data analysis. Having shown the excellent wavelength reproducibility and linearity, we will report next the analysis results for three exemplary systems, *i.e.*, two proteins F-BSA and GFP as well as AuNCs.

### Fluorescent proteins

**Fig. 7:**
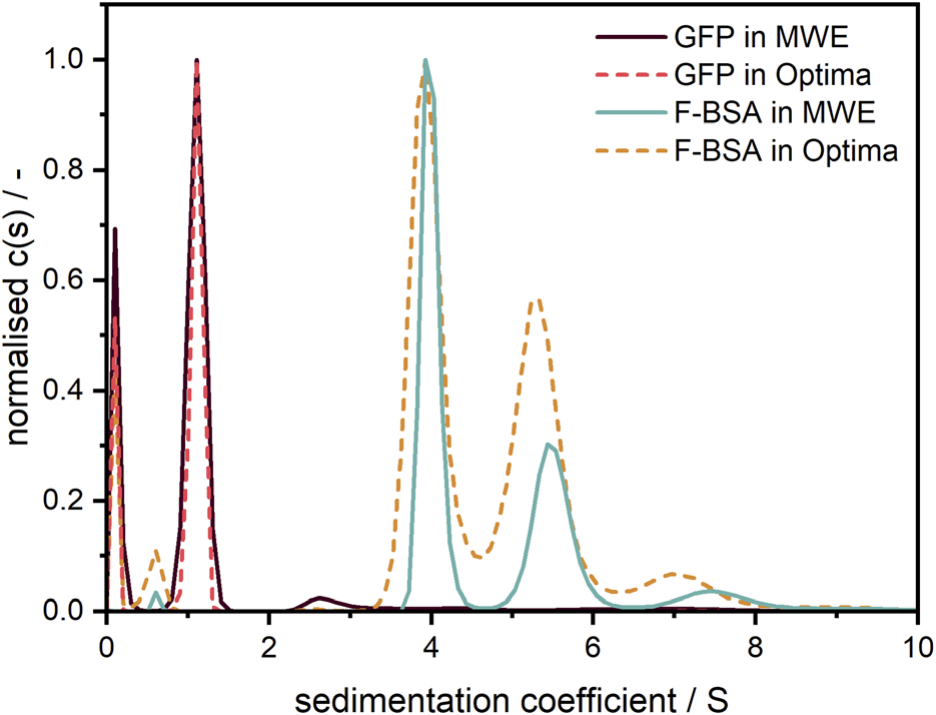
Comparison of sedimentation coefficient distributions obtained using emission (straight line) and absorbance (dotted line) detection in the Gen2 MWE-AUC and Optima AUC, respectively. The 520 nm laser was used for excitation in the MWE-AUC measurements. F-BSA was diluted in a tris (12 M)/NaCl (15 M) buffer and was measured at 0.45 μM in both devices. F-BSA data was evaluated at 550 nm for the MWE-AUC. GFP was diluted in a glycerol/PBS buffer and was measured at 2.5 μM in the MWE-AUC (data are shown for emission at 570 nm) and 5 μM in the Optima AUC. The presence of glycerol led to a comparably high solvent density (1.057 g cm^−3^) and viscosity (1.9 mPas),^22,23^ which explains the small apparent sedimentation coefficients of GFP. All protein measurements were carried out at 40 000 rpm and 20 °C. The fit results and residuals are shown in the ESI, Fig. S9.†

To demonstrate and confirm the performance of our setup, a comparison between emission and absorbance data was conducted using the Optima AUC and the Gen2 MWE-AUC, respectively. As shown in Fig. 7, both proteins exhibit nearly identical sedimentation coefficient distributions when comparing the two devices. F-BSA shows as expected its characteristic monomer-dimer-trimer structure. Interestingly, the monomer-dimer ratio differs slightly between the absorption and emission measurements, which must originate from different relative signal contributions of both species in the two detection systems (weaker fluorescence signal from the dimer). Notably, the species are baseline separated for the data retrieved by the MWE-AUC, while the Optima AUC has apparently a lower resolution (all experiments were performed at the same temperature and rotor speed; the parameters used for data analysis were identical).

In contrast to F-BSA, the sedimentation coefficient distributions for GFP show only one main peak which can be attributed to the monomer. Measurements made using the two machines overlap perfectly, which validates the accuracy of our MWE-AUC, when it comes to resolving hydrodynamic characteristics of macromolecules. The additional peak close to 0 S indicates the presence of free fluorescein and light-absorbing impurities with very little molar weight as can be observed with both detection systems.

**Fig. 8:**
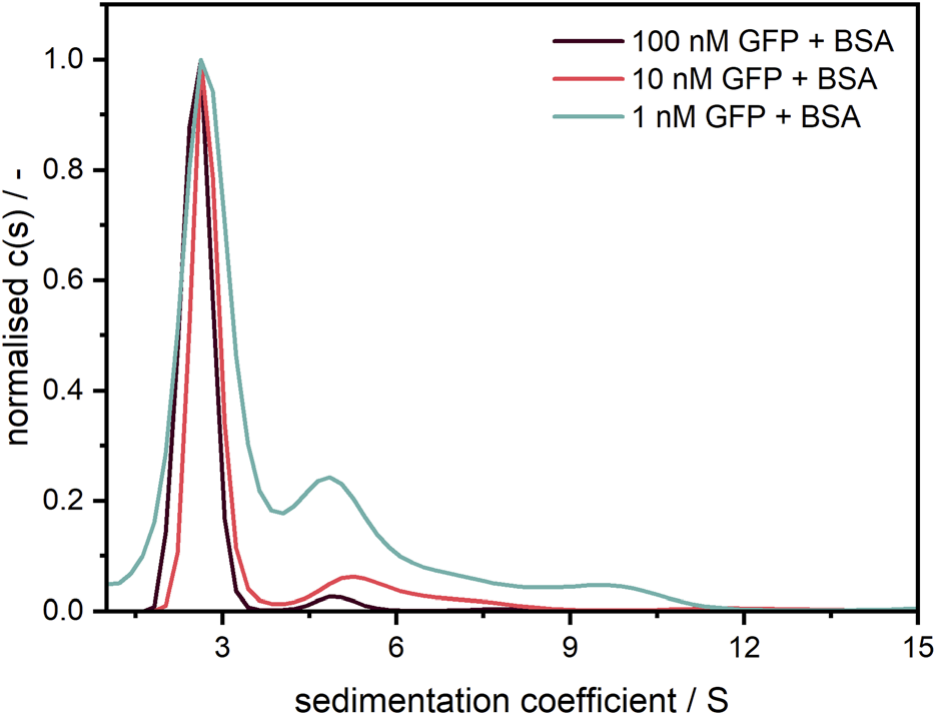
Sedimentation coefficient distributions for GFP measured at 40 000 rpm with the Gen2 MWE-AUC at several concentrations with an excitation at 405 nm. Measurement data were evaluated at 511 nm. Following similar procedure as Schuck *et al.*,^39^ GFP was diluted in a 1.5 μM BSA solution to prevent the adsorption at the cell windows.^20^

After confirming the credibility of our measurements, GFP was additionally measured at different concentrations in the nanomolar range (*cf.*Fig. 8). A good data quality and consistent sedimentation coefficients distributions are retrieved for concentrations (1–100 nM) spanning two orders of magnitude (>three orders of magnitude when considering the data shown in Fig. 7). The additional peaks at larger sedimentation coefficients might originate from agglomerates of GFP with unlabelled BSA, an effect which apparently increases at higher relative BSA concentrations.

Based on our detailed studies of the detector linearity reported above, we expect that the measurement range can be extended to much lower concentrations in the pM range. Detailed investigations of the detection limit for strongly associating systems will be followed in subsequent work.

### Gold nanoclusters

In addition to the two proteins, AuNCs were measured to demonstrate applicability of the Gen2 MWE-AUC for a particle system. Nanoscale gold particles exhibit a significantly different behaviour than the bulk material and are characterised by their size-dependent optical properties in the size range of about 2–100 nm due to the localised surface plasmon resonance effect. Ultra-small, <2 nm gold nanoparticles, also referred to as AuNCs, behave more like semiconducting quantum dots having a size-dependent band gap due to the quantum confinement effect. Such AuNCs are formed in specific numbers of metal atoms and ligands.^40,41^ For our proof-of-principle investigations, we used glutathione-stabilised AuNCs, which exhibit a photoluminescent property and are therefore ideal for highlighting and illustrating the strengths of the Gen2 MWE-AUC.

**Fig. 9:**
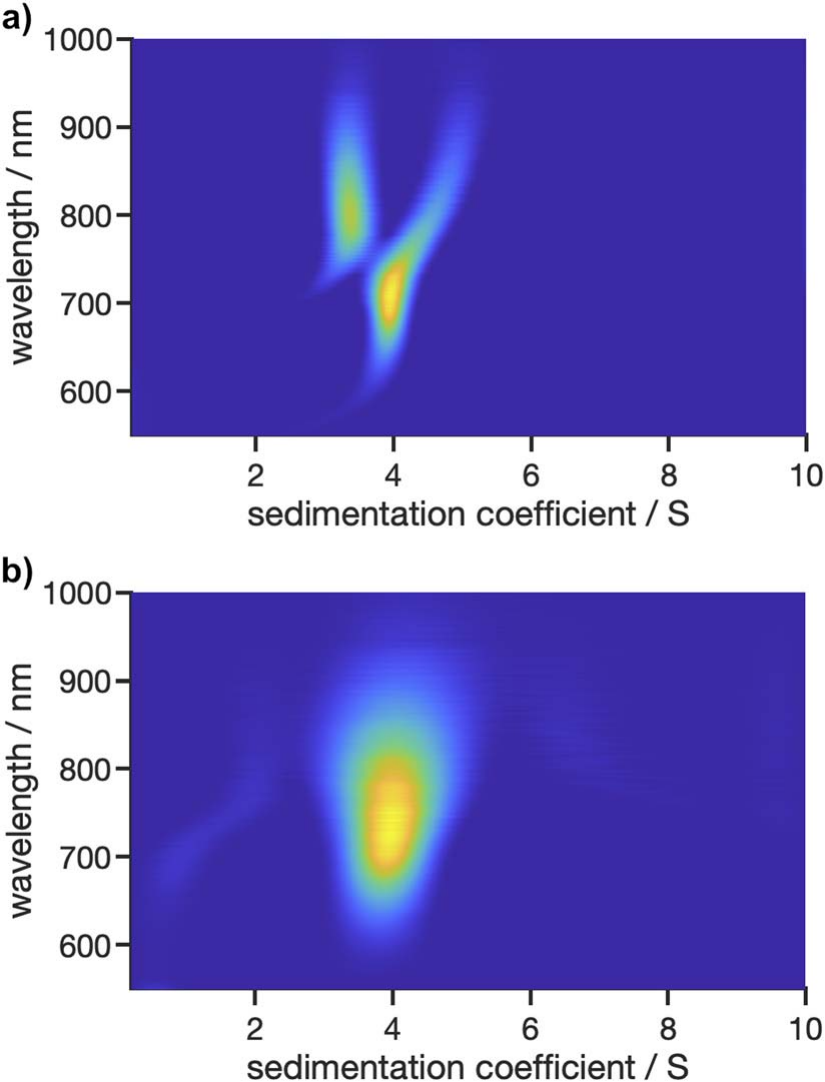
2D analysis of sedimentation velocity experiment with AuNCs performed at 40 000 rpm and 20 °C using two different excitation lasers with wavelengths of (a) 405 nm and (b) 520 nm.

As shown in Fig. 9, the glutathione-stabilised AuNCs were measured at the two different excitation wavelengths. The derived 2D sedimentation coefficient–wavelength distributions are clearly different for the two excitation wavelengths and indicate that the sample contains a variety of AuNCs with different composition having respectively different absorption and fluorescent properties. Depending on the excitation wavelength, different species contribute to the overall fluorescence signal, which is resolved by the underlying sedimentation coefficients of the different species. Two species are apparent with sedimentation coefficients of 3.4 S and 3.9 S for an excitation at 405 nm, while only one species with a sedimentation coefficient of 4.0 S is observed for an excitation at 520 nm. The corresponding emission maxima are retrieved at about 800 nm, 705 nm and 740 nm for the 3.4 S, 3.9 S and 4.0 S species, respectively. Overall, the size-dependent fluorescence of AuNCs is a well-known property which can be resolved by our Gen2 MWE-AUC in a single experiment.

For the measurement at an excitation at 405 nm, an artefact shift for the 3.9 S species towards larger sedimentation coefficient is apparent as soon as the 3.4 S is observed at longer than 750 nm wavelengths. A possible explanation for this phenomenon is the fact that the data was analysed with a fixed frictional ratio and partial specific volume, while both species should have different shapes as well as densities due to the altering gold–glutathione composition.^21^ As the contribution of each species to the overall signal is wavelength dependent, this can induce shifts in the sedimentation coefficient distributions. This is supported by the observation that the wavelength shifts can be eliminated when the frictional ratio and partial specific volume are optimised manually instead of using the fixed frictional ratio of 1 and best-fit values for the partial specific volume (*cf.* Fig. S10a†). In addition, our hypothesis is supported by a global analysis at three selected wavelengths (700 nm, 760 nm, 825 nm) using the single species model in SEDPHAT. The analysis with three discrete species provided sedimentation coefficients of 3.35 S, 3.97 S and 4.82 S, which are in line with the findings from the multiwavelength analysis.

Besides, the comparably broad distribution observed for the excitation at 520 nm indicates that there are presumably more than one species present, which would also explain the shift in the emission maximum when comparing the species to the one found at 3.9 S for the excitation at 405 nm. Multiple species with only slightly varying sedimentation coefficients are hardly distinguishable when using second derivate regularisation as it is currently implemented in our 2D analysis procedure. Remarkably, at least two species are observed when considering the non-regularised 2D distribution (*cf.* Fig. S10b†). Therefore, future work needs to focus on extending the capabilities of data analysis, *e.g.*, by implementing different regularisation schemes such as maximum entropy or Monte Carlo analysis, or by making use of established algorithms which permit altering partial specific volumes or frictional ratios as a function of the sedimentation coefficient such as the c(s, ff0)^42^ or discrete species method implemented in SEDFIT or the 2DSA^43^ and PCSA^44^ techniques implemented in UltraScan3, to allow for an in-depth multidimensional analysis of structure–property relationship of AuNCs using MWE-AUC.

## Conclusions

An improved MWE-AUC was developed and is presented in this paper. Essential modifications of mechanical design, optical scheme, and data acquisition software together with the use of a high-performance spectrograph and an EMCCD camera permit significant improvements for spectral possibilities of the setup and the quality of the data obtained, especially due to the reduction of systematic signal noise and increased sensitivity. The most important developments constitute the excitation of sample fluorescence by either 405 nm or 520 nm lasers, a set of three switchable tuneable gratings (150/600/1800 L mm^−1^) in the spectrograph, allowing optimisation of spectral resolution in an order of magnitude range, the systematic assessment of inner filter effects using a motorised vertical translation stage, and new mono-sector cells featuring a 3 mm titanium centrepiece for extended chemical compatibility. The signal amplitude linearity of our detection system was confirmed for more than five orders of magnitude and the dependence of the signal-to-noise ratio on signal amplitude was studied systematically. The performance and applicability of the MWE-AUC was further demonstrated by validation of sedimentation coefficient distributions for biological molecules F-BSA and GFP, and the correlation of sedimentation coefficient distributions and fluorescence spectra for AuNCs.

Being complementary to other AUC detection systems, our ready-for-use Gen2 MWE-AUC, being to our knowledge the first and sole one of its kind, constitutes an important next step for the multidimensional characterisation of complex colloids, now also comprising size-, shape- and composition-dependent multiwavelength emission properties. Future work will thus mainly target the systematic and in-depth investigations of colloids making use of the manifold possibilities of the Gen2 MWE-AUC. This includes the investigation of the structure–property relations of particles and macromolecules including quantum dots and AuNCs.

To make use of the new possibilities offered by the MWE-AUC, current data processing algorithms must be upgraded to include all the specific features of the measurement data and of the samples to be investigated. Particularly, the dependence of the fit parameters on the sedimentation coefficient value, especially of the partial specific volume and frictional ratio, must be included to properly analyse strongly interacting macromolecules and particle interactions with specific optical footprints.

Global analysis schemes will give rise to more efficient multiwavelength analysis and might even allow coupling of data from different detection systems for deeper insights into structure–property relationships of colloids such as the size-dependent fluorescence quantum yield of quantum dots. For such investigations, possibility to measure the sample extinction at the excitation wavelength simultaneously with fluorescence would be very useful. Importantly, the optical efficiency of the MWE-AUC needs to be evaluated to retrieve quantitative data from such investigations. Besides, there are still several interesting subjects for further MWE-AUC performance improvements such as the increase of the signal-to-noise ratio by a decrease of the digitalisation rate or the use of on-sensor pixel binning over a large area as well as activation of electron multiplication in the EMCCD camera. The latter is important, when Raman detection is targeted, a possibility which is in principle available with our setup. A fast variable optical attenuator with computer software control would allow the setting of sample-specific excitation power for extending the concentration range. Finally, multiwavelength sample excitation could be used to study complex systems of particles with different absorption spectra.

## Data availability

The data supporting the findings of the work reported in this manuscript are available on Zenodo, DOI: https://10.5281/zenodo.8388976.

## Author contributions

VL: data curation, formal analysis, investigation, validation, visualisation, writing and editing. GO: conceptualisation, data curation, formal analysis, investigation, methodology, validation, visualisation, writing and editing. SW: conceptualisation, data curation, formal analysis, investigation, methodology, validation. UF: conceptualisation, data curation, formal analysis, investigation, review. LH: data curation, formal analysis, investigation, validation, visualisation. WP: conceptualisation, funding acquisition, project administration, resources, supervision, review and editing. JW: conceptualisation, data curation, formal analysis, funding acquisition, methodology, project administration, resources, software, supervision, validation, visualisation, writing, review, and editing.

## Conflicts of interest

There are no conflicts to declare.

## Supplementary Material

NA-006-D3NA00980G-s001
