# Supplementary material for: Development of an advanced multiwavelength emission detector for the analytical ultracentrifuge
Source: Nanoscale Adv. 2024 Feb 19;6(10):2611–22. doi: 10.1039/d3na00980g (PMC11093262; doi:10.1039/d3na00980g)
Supplement: NA-006-D3NA00980G-s001 [file NA-006-D3NA00980G-s001.pdf]

Electronic Supplementary Material (ESI) for Nanoscale Advances.
This journal is © The Royal Society of Chemistry 2024

Electronic Supplementary Information

Development of an advanced multiwavelength emission detector for the analytical ultracentrifuge

Vanessa Lautenbach,[a] Georgy Onishchukov,[a,b,c] Simon Wawra,[a,b,d] Uwe Frank,[a,b,e] Lukas Hartmann,[a] Wolfgang Peukert,[a,b] Johannes Walter[a,b]

[a.] Institute of Particle Technology (LFG), Friedrich-Alexander-Universität Erlangen-Nürnberg (FAU), Cauerstraße 4, 91058 Erlangen, Germany. E-mail: johannes.walter@fau.de
[b.] Interdisciplinary Center for Functional Particle Systems (FPS), Friedrich-Alexander-Universität Erlangen-Nürnberg (FAU), Haberstraße 9a, 91058 Erlangen, Germany.
[c.] Max Planck Institute for the Science of Light, Staudtstraße 2, 91058 Erlangen, Germany.
[d.] Current affiliation: Boehringer Ingelheim Pharma GmbH & Co. KG, Innovation Unit, Analytical Development Biologicals, Birkendorfer Straße 65, 88400 Biberach an der Riß, Germany.
[e.] Current affiliation: Carl Zeiss Vision GmbH, Turnstraße 27, 73430 Aalen, Germany.

Hardware Modifications

Table T1. *Components of the Gen2 MWE-AUC.*

Identifier	Description	Supplier	Model
**Optical components**			
M1	Ø1/2" 90° off-axis parabolic mirror, RFL = 15 mm, protected silver coating	Thorlabs	MPD00M9-P01
M2	Ø1" 90° off-axis parabolic mirror, RFL = 6", protected silver coating	Thorlabs	MPD169-P01
M3	Ø2" 90° off-axis parabolic mirror with Ø3 mm hole, RFL = 4", protected silver coating	Thorlabs	MPD249H-P01
M4	Ø3" 45° off-axis parabolic mirror, RFL = 6", protected silver coating	Thorlabs	MPD364-P01
Spectral filter	Ø1" longpass filter, 520 nm cut on, OD > 3 at 520 nm	Omega	525ALP
Spectral filter*	Ø1" longpass filter, 410 nm cut on, OD > 6 at < 407nm, T_{avg} > 93% for 412 - 918 nm	Semrock	LP02-407RU-25
Laser 520 nm	Diode laser, 80 mW TEM00, center wavelength 520 nm, bandwidth ~1 nm, with temperature control and digital amplitude modulation	Integrated Optics	0520L-11A-NI-NT-NF
Laser 405 nm*	Diode laser, 180 mW TEM00, center wavelength 405 nm, bandwidth ~1 nm, with temperature control and digital amplitude modulation	Integrated Optics	0405L-11A-NI-NT-NF
Spectral filter*	Ø1/2" Nanopede 520/15 bandpass filter, 520 nm center, 20 nm bandwidth, T_{avg} > 93% for 512.5 – 527.5 nm, OD > 5 in 536 – 900 nm region	Semrock	FBP01-520/15-12.5
Spectral filter*	Ø1/2" Nanopede 400/16 bandpass filter, 400 nm center, 20nm bandwidth, T_{avg} > 93% for 392 – 408 nm, OD > 5 for 415 – 900 nm	Semrock	FBP01-400/16-12.5
Variable attenuator*	Ø50 mm continuously variable neutral density disk filter, OD: 0.04 - 2.0, 350 - 700 nm antireflection coating	Thorlabs	NDC-50C-2-A with NDC-PM

**Mechanical components** (only these in the chamber)			
Fiber adapter	Ø1/2" FC/APC fiber adapter plate	Thorlabs	SM05FCA
Fiber cage adapter	30 mm to 16 mm cage-system right-angle adapter	Thorlabs	SP30
M1 mount	Ø1" kinematic cage-compatible mount	Thorlabs	KC1/M
M1 adapter	Ø1" adapter for Ø1/2" off-axis parabolic mirrors	Thorlabs	MP127P1
M2 mount	Right-angle kinematic Ø1" off-axis parabolic mirror mount for 30 mm cage system	Thorlabs	KCB1P/M
M3 mount	Ø2" precision kinematic mirror mount	Thorlabs	KS2
Fiber mount	Ø1" 5-axis kinematic mount	Thorlabs	K5X1
Fiber adapter	Ø1" FC/PC fiber adapter plate	Thorlabs	SM1FC
Cage rods	Cage assembly rod	Thorlabs	ER6
Radial linear translation stage	TravelMax translation stage, 25 mm travel, crossed-roller bearings, 20 kg load	Thorlabs	LNR25M/M
Linear actuator	Low-vacuum linear step-motor actuator with built-in controller, 25 mm travel, 0.048 µm step size, 1 µm repeatability	Zaber	T-NA-08A-24-SV2
Vertical linear translation stage*	Low-vacuum vertical translation stage with step motor, 20 mm travel, 0.095 µm step size, 1 µm repeatability	Zaber	VSR20A-V1T4-MC03
**Vacuum components**			
Fiber feedthrough	Single-mode QS-488 fiber (3.5/125/900 µm) with adjustable FC/APC connectors and multi-mode QM-UVVIS fiber* (200/125/900 µm, NA 0.22) with FSMA905 connectors	OZ Optics	
Step-motor cable feedthrough	18 wires	Lemo	SWH.3S.318.CLLDV
Light-barrier cable feedthrough	4 wires	Lemo	EVP.0V.304.CLLSV
**Electronic components**			
Light barrier	Reflective photosensor		RPR-220
Control computer card	Multifunctional analog/digital I/O card (3 counters are used)	National Instruments	PCIe-6321
Controller for vertical translation stage*	X-Series stepper motor controller	Zaber	X-MCA-KX13AF
Diffraction grating spectrograph*	High-performance imaging spectrograph with interchangeable triple grating turret, focal length 300mm, f/3,9 aperture ratio, 150 l/mm, 600 l/mm and 1800 l/mm gratings with 500 nm blaze wavelength	Teledyne Princeton Instruments	HRS-300MS-NI
EMCCD camera*	ProEM+: 16002 eXcelon3 back-illuminated EMCCD camera, 1600 X 200, 16 µm pixels, -75°C sensor cooling, 16 bit 8 MHz ADC	Teledyne Princeton Instruments	PRO1600x200BX3
CMOS camera*	Monochrome camera with Sony IMX183 sensor, 1”, 20.5MP, 2.2µm pixels	Matrix Vision	BF3-5M-0205ZG-112510

** New components in the Gen2 MWE-AUC*

Hardware Specifications

Table T2. *Specifications of the Gen2 MWE-AUC and comparison with the AU-FDS.*

**Parameter / Option**		**Gen2 MWE-AUC**	**AU-FDS**
Sample volume[1], µl	1.5 mm double-sector	50[1a]	
	3 mm mono-sector	200	
Excitation laser, nm		405 or 520, external[2]	various options, built-in
Excitation power, mW		≤ 20[3]	various options
Tuneability of spectral range		single grating, mid-focal length imaging spectrograph[4]	n.a., spectral filter
Spectral range, nm	grating 150 l/mm	~540, e.g., 400 – 937	fixed, e.g., 505 - 565 for 488nm excitation laser
	grating 600 l/mm	~130, e.g., 515 – 643	
	grating 1200 l/mm	~35, e.g., 525 – 558	
Spectral resolution, nm	grating 150 l/mm	2.0[5]	n.a.
	grating 600 l/mm	0.5	
	grating 1200 l/mm	0.14	
Detector		scientific-grade EMCCD camera	photomultiplier
Signal dynamic range, logs		~5<br>16 bit ADC & 1-2-4 gain[6]	ADC & tuneable PMT gain

[1] Required volume for sedimentation velocity experiments to provide 85-90% cell filling with well visible meniscus. For sedimentation equilibrium using special centrepieces, required sample volume can be up to 4 times smaller.

[1a] Estimated reduction of the signal amplitude of a 1.5 mm compared to 3 mm centrepiece is ~ 20%.

[2] External laser can be easily exchanged by the operator. There are no limitations on the laser dimensions, power consumption, cooling, etc.

[3] Variable optical attenuator allows tuneable power reduction by two orders of magnitude without changing other parameters of the laser.

[4] External spectrograph, having no limitation on its size, provides a unique flexibility of spectral range and spectral resolution by using different gratings (three on a turret with easy exchange of turrets by the operator). The central wavelength is quickly tuneable also during measurements by changing the grating angle using computer control, as well as the range by changing the grating used. In addition to the EMCCD camera, a second, e.g., NIR or UV-optimized camera, photomultiplier or any other detector can be installed on the second spectrograph output with computer control of the output used.

[5] Resolution can be improved by postprocessing of the 2D camera image, as well as by reducing the tuneable entrance slit with corresponding reduction of the signal amplitude.

[6] Without electron multiplication (EM). The dynamic range can be further increased by integration over several camera pixels. EM gain up to x1000 allows single photon counting.

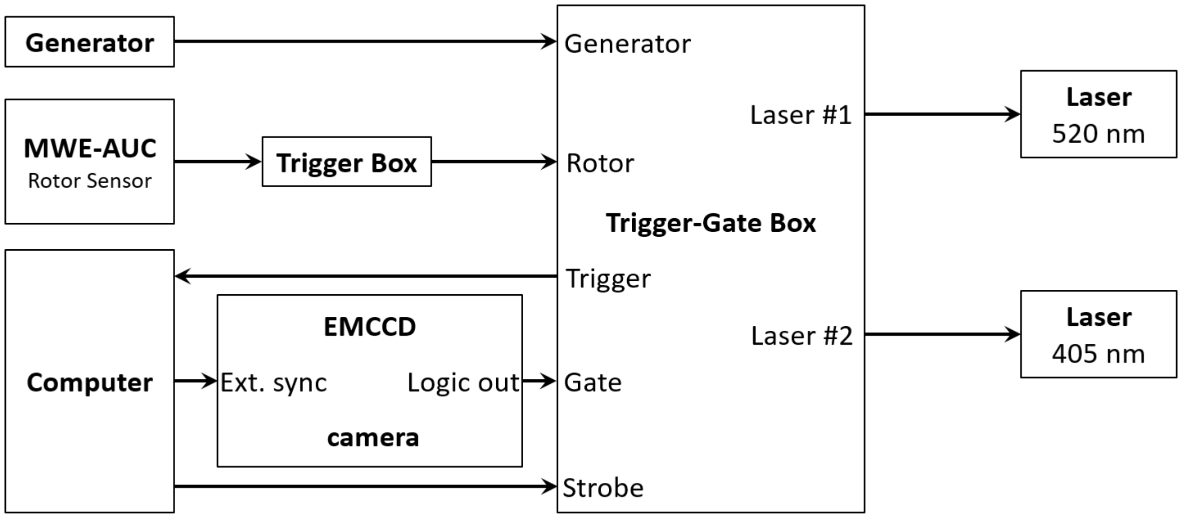

Figure S1. *Block scheme of the triggering.*

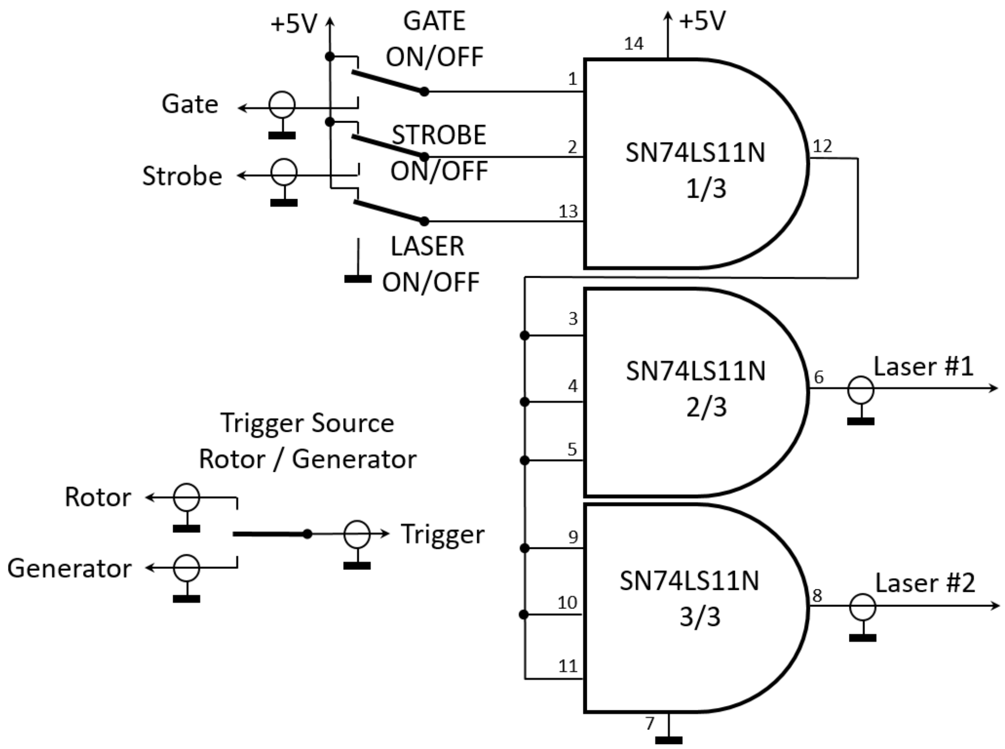

Figure S2. *Electrical scheme of trigger-gating box.*

Supplementary data

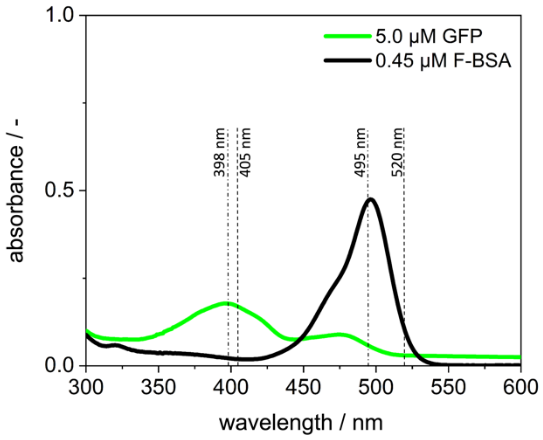

Figure S3. *Absorbance spectra of GFP and F-BSA measured prior to AUC measurements with a Varian Cary 100 spectrophotometer (2.0 nm slit width, 10 mm optical path, 1.0 nm spectral resolution). The respective absorption maxima are at 398 nm and 495 nm for GFP and F-BSA, respectively. Dashed lines mark the 398 nm and 495 nm used for the absorbance detection in the Optima AUC as well as the 405 nm and 520 nm excitation laser lines used in the Gen2 MWE-AUC.*

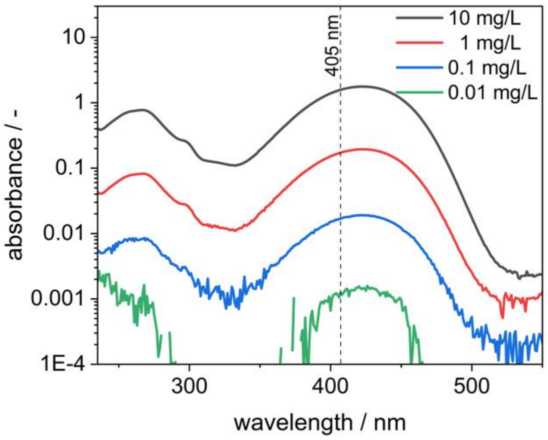

Figure S4*. Absorbance spectra for different concentrations of Coumarin 153 measured prior to AUC measurements with a Varian Cary 100 spectrophotometer (2.0 nm slit width, 10 mm optical path, 1 nm spectral resolution). Dashed line marks the 405 nm excitation laser line.*

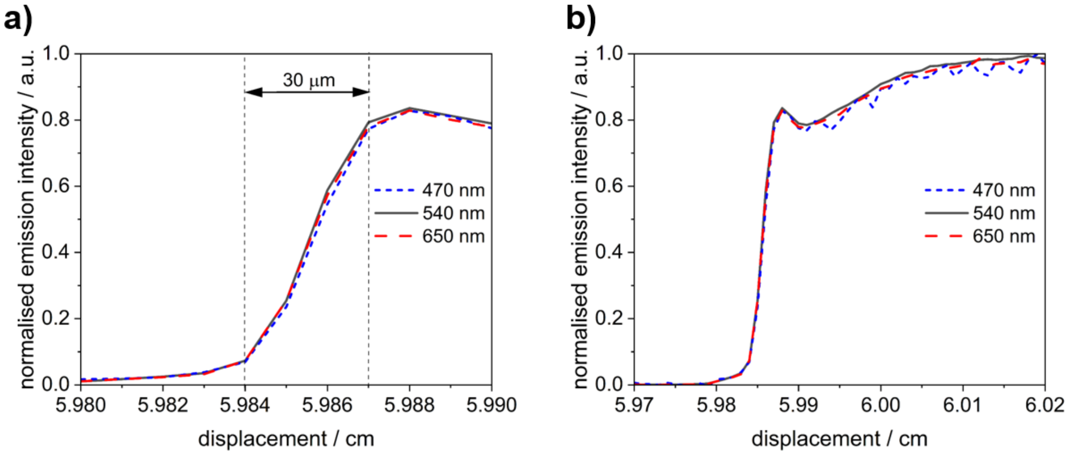

Figure S5. *Radial scan of 0.1 mg/L Coumarin 153 dissolved in ethanol showing the meniscus for various wavelengths. Data are recorded with 10 µm steps at 5,000 rpm. The 405 nm laser was used for excitation, which provides a focal spot smaller than the 520 nm laser. a) Radial resolution of 30 µm as the distance of the edge rise from the 10 % to the 90 % level. b) Distortions of the sedimentation profile in the meniscus region.*

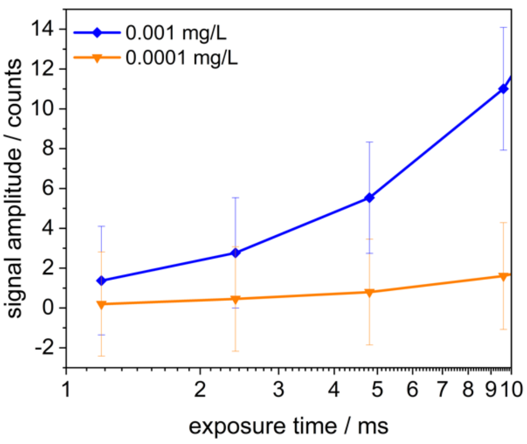

Figure S6. *Signal amplitude of Coumarin 153 samples diluted in ethanol to target concentrations of 0.001 mg/L and 0.0001 mg/L measured at different exposure times to illustrate the linearity of the Gen2 MWE-AUC. Other than in Figure 4a) in the main manuscript, the linear y-axis allows a proper visualisation of error bars for very low signal amplitudes.*

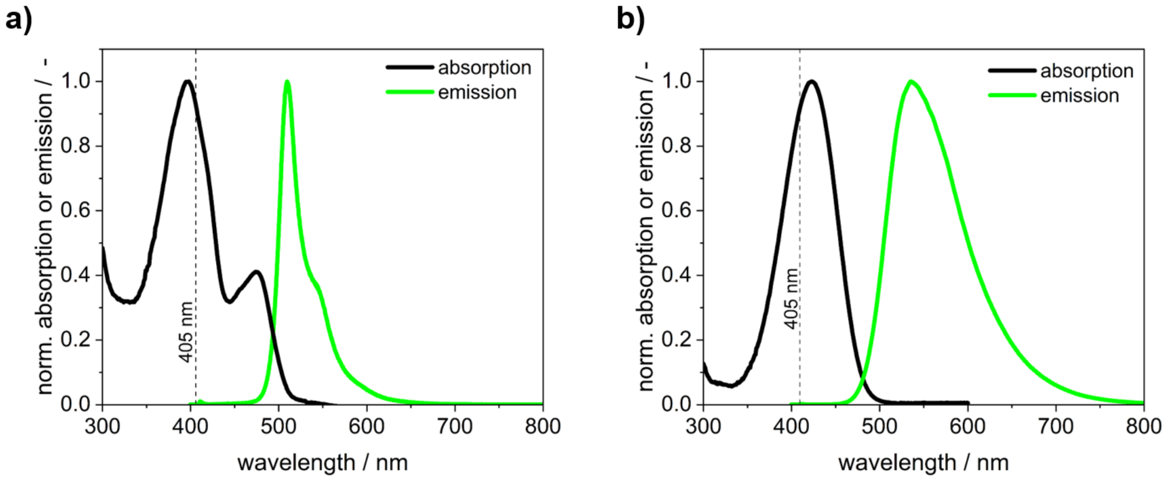

Figure S7. *a) Normalised absorption and emission spectra of GFP diluted in a 20 v% glycerol/PBS buffer. Absorption and emission are shown for GFP concentrations of 5 µM and 2.5 µM, respectively. b) Normalised absorption and emission spectra for 1 mg/L Coumarin 153 dissolved in ethanol. Absorption spectra were measured with Varian Cary 100 spectrophotometer (2.0 nm slit width, 10 mm optical path, 1 nm spectral resolution). Dashed lines mark the 405 nm excitation laser line.*

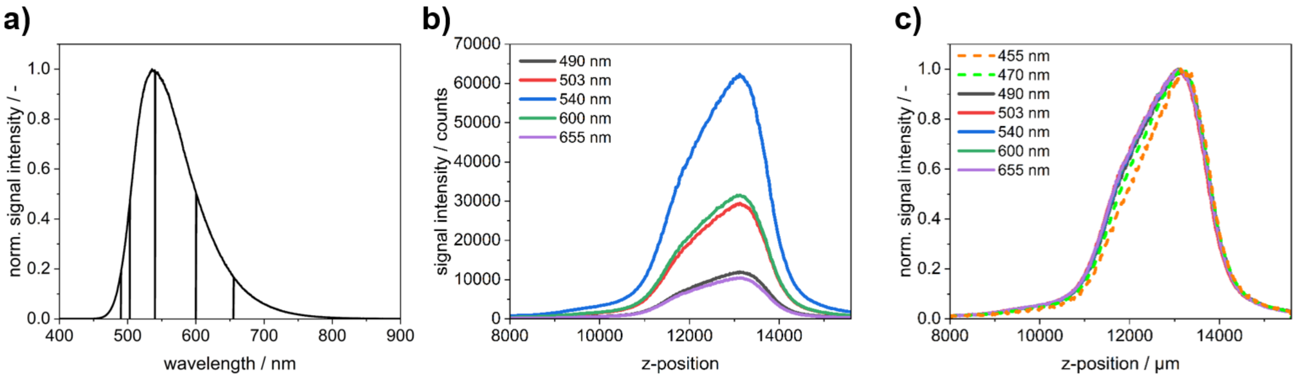

Figure S8. *a) Emission spectrum of 10 mg/L Coumarin 153 dissolved in ethanol recorded at z-position 12,500 µm. Straight lines indicate the selected wavelengths plotted in b) and c). b) Z-scans for the wavelengths shown in a) demonstrating distortions by the inner filter effects. c) Normalised z-scans of Coumarin 153 at several wavelengths, demonstrating the wavelength independence of the Gen2 MWE-AUC. The asymmetry visualises the apparent inner filter effects. Dashed lines show the z-scans for 455 nm and 470 nm demonstrating additionally the weak but still apparent secondary inner filter effect.*

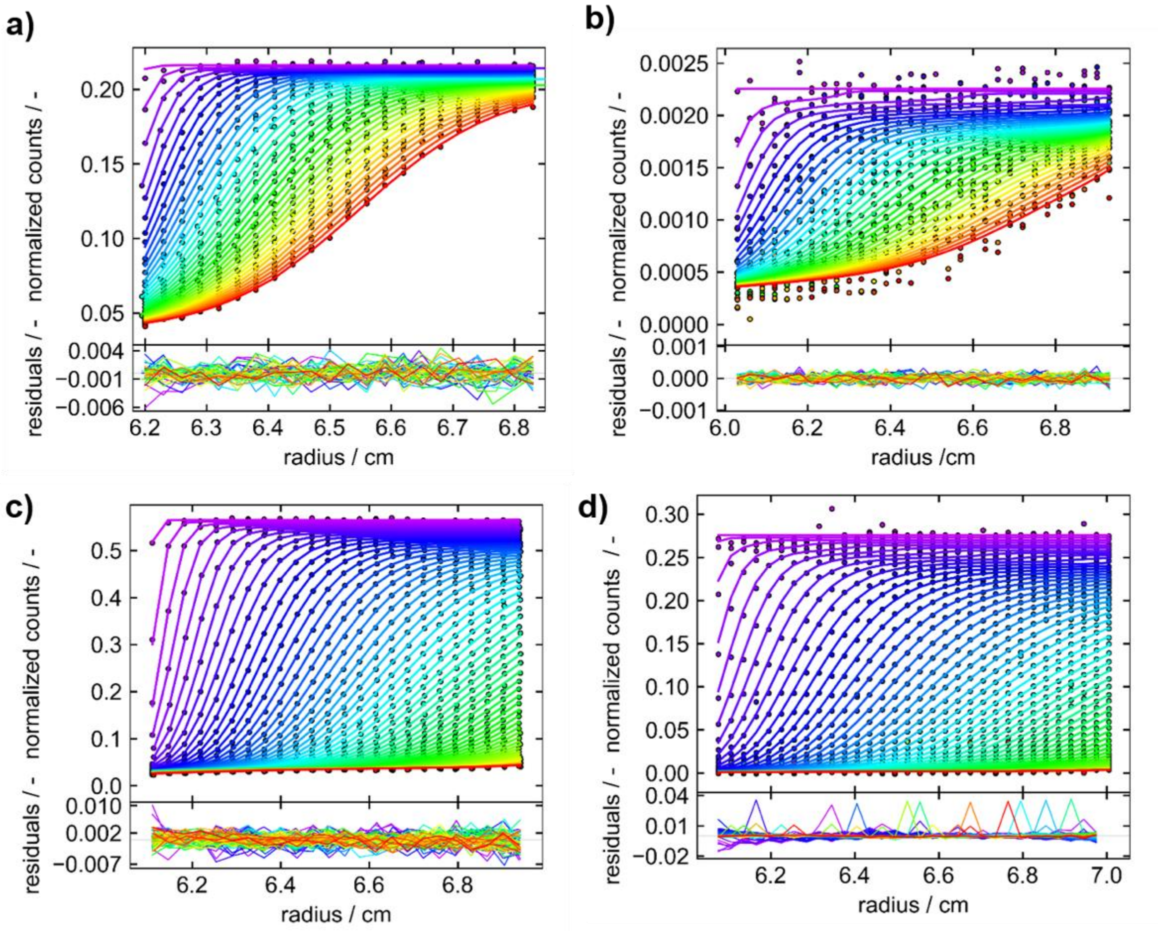

Figure S9. *Fit results and residuals for GFP (a, b) and F-BSA (c, d) measured with the Optima AUC with the absorption detection system (a, c) and Gen2 MWE-AUC (b, d). The first 350 scans were considered for evaluation. The 520 nm laser was used for excitation in the MWE-AUC measurements. GFP was diluted in a glycerol/PBS buffer and was measured at 5 µM in the Optima AUC and 2.5 µM in the MWE-AUC (data shown are for emission at 570 nm). The comparably slow sedimentation of GFP can be explained by the presence of glycerol in the sample, whose density and viscosity are increased compared to water. F-BSA was diluted in a Tris (12 mM)/NaCl (15 mM) buffer and was measured at 0.45 µM in both devices. F-BSA data by the MWE-AUC were evaluated at 550 nm. Note: Noise spikes observable in d) are due to "cosmic rays" being recorded by the EMCCD camera. Such environmental pollution only occurs sporadically and can be corrected for during data processing. Here the data were not post-treated and are plotted as recorded. All protein measurements were carried out at 40,000 rpm and 20°C.*

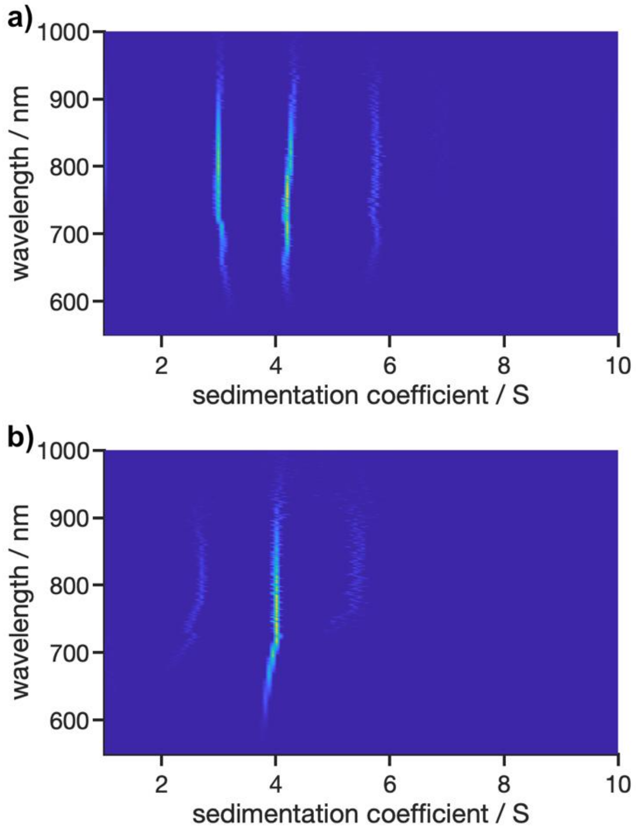

Figure S10. *2D analysis of sedimentation velocity experiments of AuNCs performed at 40,000 rpm, 20°C and an excitation at a) 405 nm and b) 520 nm. Data were integrated over 1.5 nm spectral bandwidth. For a) a partial specific volume of 0.65 cm^3/g and frictional ratio of 1.4 was used for fitting the data instead of the best-fit values (0.547 cm^3/g and 1.0, respectively) used for deriving the distributions shown in Figure 9a in the main manuscript. Both values were chosen manually in such way that the wavelength shift for each species is as small as possible. For b) the best-fit values for the partial specific volume (0.63 cm^3/g) and frictional ratio (1.0) were used as no pronounced wavelength shift was observed, which is identical to the results reported in Figure 9b in the main manuscript. Both figures shown here display the non-regularised distributions.*

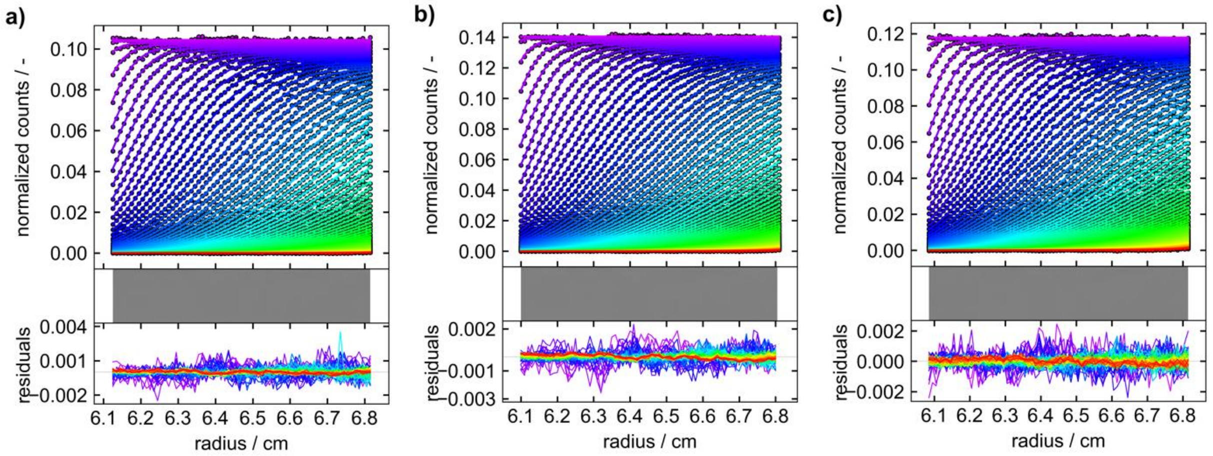

Figure S11. *Fit results for sedimentation profiles and residuals of global analysis of AuNCs in SEDPHAT at wavelengths of a) 700 nm with bandwidth 5 nm, b) 760 nm with bandwidth 10 nm, and c) 825 nm with bandwidth 5 nm. The excitation was at 405 nm. Sedimentation coefficients of 3.35 S, 3.97 S and 4.82 S were obtained.*
